# Applying graph theory to protein structures: an Atlas of coiled coils

**DOI:** 10.1093/bioinformatics/bty347

**Published:** 2018-05-02

**Authors:** Jack W Heal, Gail J Bartlett, Christopher W Wood, Andrew R Thomson, Derek N Woolfson

**Affiliations:** 1 School of Chemistry, University of Bristol, Bristol, UK; 2 School of Chemistry, University of Glasgow, Glasgow, UK; 3 School of Biochemistry, University of Bristol, Bristol, UK; 4 BrisSynBio, University of Bristol, Life Sciences Building, Bristol, UK

## Abstract

**Motivation:**

To understand protein structure, folding and function fully and to design proteins *de novo* reliably, we must learn from natural protein structures that have been characterized experimentally. The number of protein structures available is large and growing exponentially, which makes this task challenging. Indeed, computational resources are becoming increasingly important for classifying and analyzing this resource. Here, we use tools from graph theory to define an Atlas classification scheme for automatically categorizing certain protein substructures.

**Results:**

Focusing on the α-helical coiled coils, which are ubiquitous protein-structure and protein–protein interaction motifs, we present a suite of computational resources designed for analyzing these assemblies. iSOCKET enables interactive analysis of side-chain packing within proteins to identify coiled coils automatically and with considerable user control. Applying a graph theory-based Atlas classification scheme to structures identified by iSOCKET gives the Atlas of Coiled Coils, a fully automated, updated overview of extant coiled coils. The utility of this approach is illustrated with the first formal classification of an emerging subclass of coiled coils called α-helical barrels. Furthermore, in the Atlas, the known coiled-coil universe is presented alongside a partial enumeration of the ‘dark matter’ of coiled-coil structures; i.e. those coiled-coil architectures that are theoretically possible but have not been observed to date, and thus present defined targets for protein design.

**Availability and implementation:**

iSOCKET is available as part of the open-source GitHub repository associated with this work (https://github.com/woolfson-group/isocket). This repository also contains all the data generated when classifying the protein graphs. The Atlas of Coiled Coils is available at: http://coiledcoils.chm.bris.ac.uk/atlas/app.

## 1 Introduction

With more than 130 000 structures currently available in the Protein Data Bank (PDB) (Berman, 2000), the need for protein-structure classification is clear (Andreeva *et al.*, 2014; Sillitoe *et al.*, 2015). Such classifications demonstrate the structural diversity exhibited by proteins in nature; develop our understanding of proteins; and facilitate comparisons between structures. Further, protein-structure classifications provide inspiration for protein designers to identify the structures that are not yet present in these schemes and then construct them *de novo* (Kuhlman *et al.*, 2003; Michalopoulos *et al.*, 2004; Thomson *et al.*, 2014; Zaccai *et al.*, 2011). However, the ever-increasing deposition rate of new structures into the PDB puts considerable pressure on classification schemes to be fully automated to remain up-to-date and to be truly useful.

Classification schemes for protein folds include SCOPe (Andreeva *et al.*, 2014), CATH (Sillitoe *et al.*, 2015) and ECOD (Cheng *et al.*, 2014), which combine expert curation with automated methods. These are hierarchical, with individual proteins assigned membership to one of many different nested categories. The TOPS database (Michalopoulos *et al.*, 2004) provides cartoon visualization aids for inspecting and comparing protein folds, which inspired the design of the *de novo* protein fold Top7 (Kuhlman *et al.*, 2003).

Gaps in these schemes represent what has been termed the ‘dark matter’ of protein space (Taylor *et al.*, 2009; Woolfson *et al.*, 2015); that is, those structures that are theoretically possible but have yet to be observed in nature. One problem with existing classifications is that the gaps are generally difficult to define; i.e. how do we enumerate the dark matter? We sought a classification scheme for existing structures and the dark matter, as well as means that could delineate them. The system we have designed is fully automated, and its basis in mathematical graph theory is general enough that it can be readily applied to a diverse set of protein motifs.

Herein, we have applied our classification scheme to the ubiquitous folding motif of the α-helical coiled coil, which none of the above classification schemes deal with despite coiled coils being present in up to 10% of all eukaryotic proteins (Liu and Rost, 2001; Rackham *et al.*, 2010). To address this, we used an alternative method for classification, which emulates the Periodic Table in structure (Moutevelis and Woolfson, 2009). This is similar to approaches used by others for classifying secondary structure combinations in proteins and protein complexes (Ahnert *et al.*, 2015; Taylor, 2002).

**Fig. 1.**
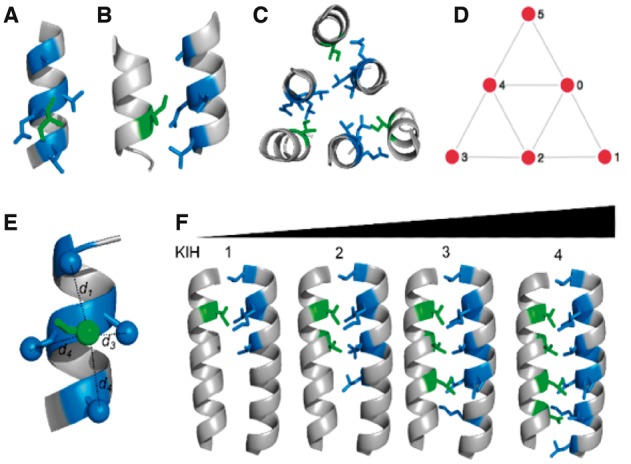
From knobs-into-holes (KIH) interactions to coiled coils to simple mathematical graphs. (**A, B**) Orthogonal views of a KIH interaction. The side chain of the knob residue (green) projects into the hole formed by the side chains of four residues (blue) on another helix. (**C**) An arrangement of six helices interacting in three pairs via KIH interactions. The structure shown is part of the core structure of the envelope glycoprotein GP2 from Ebola virus (PDB: 2ebo). (**D**) Simplified representation of all the KIH interactions in the structure as a mathematical graph. Nodes (red circles) represent the helices, and edges linking the nodes (grey lines) represent KIH packing between the associated helices. The KIH interactions in (C) form part of the edges 1→2, 3→4, 5→0. (**E, F**) Thresholds used to define edges in the Atlas Classification: (E) The SOCKET cut-off distance, scut, is a user-defined maximum for distances d_1_ through d_4_ between the centres of mass of the side chains that define the hole and that of the knob residue needed to constitute a KIH interaction. (F) A pair of interacting helices must have a total KIH interactions of >kcut. Images (A–C, E, F) were generated using PyMOL (www.pymol.org) (Color version of this figure is available at *Bioinformatics* online.)

Coiled coils comprise two or more α helices that pack tightly together via interdigitation of side chains in a geometry known as knobs-into-holes (KIH) packing (Fig. 1) (Crick, 1953; Lupas and Gruber, 2005; Woolfson *et al.*, 2012). A knob is a side chain projecting from one helix that packs into the hole formed by four side chains on an adjacent helix. Extended regions of KIH packing cement the core of a coiled coil, locking hydrophobic faces of amphipathic helices together away from solvent. The program SOCKET (Walshaw and Woolfson, 2001) finds KIH interactions within protein structures and, therefore, can identify coiled coils automatically. Application of SOCKET to the PDB has delivered the CC+ database (http://coiledcoils.chm.bris.ac.uk/ccplus/search/) (Testa *et al.*, 2009), from which the Periodic Table of Coiled Coils (PTCC) (Moutevelis and Woolfson, 2009) has been manually curated. However, the number of structures in the PDB has more than doubled since the PTCC was introduced and an update is overdue. Furthermore, and importantly for protein design, the ‘dark matter’ of coiled coils is not explicitly defined in the PTCC, making it difficult to identify the next design challenges. For example, there are clear gaps in the first row of the PTCC beyond the classical and abundant coiled-coil dimers, trimers and tetramers; though these gaps are being filled to some extent through the *de novo* design of so-called α-helical barrels, which have 5 or more α helices arranged about a central super-helical axis (Thomson *et al.*, 2014; Woolfson *et al.*, 2012; Zaccai *et al.*, 2011).

As is evident from foregoing manual inspection and curation of coiled-coil structures (Lupas and Gruber, 2005) and from CC+ and the PTCC, coiled coils are abundant and take on a variety of structural forms. These range from the relatively simple coiled-coil dimers (Lupas and Bassler, 2017; Woolfson, 2017), through more-complicated assemblies such as the 12-helix barrel of TolC (Koronakis *et al.*, 2000) and to the ‘trimer of hairpins’ of many viral glycoproteins (Chan *et al.*, 1997; Malashkevich *et al.*, 1999; Walshaw and Woolfson, 2003). This diversity of structure corresponds to a diversity of function, with the example coiled-coil structures above being involved in DNA binding and transcriptional control in eukaryotes, export mechanisms from bacterial cells, and virus-host membrane fusion, respectively.

Here, to automate the recognition and classification of the diverse CC structures, we turn to mathematical graphs, which are used to represent pairwise interactions within sets of objects. The objects form the nodes of the graphs (which for coiled coils are the α helices), and the interactions between them form its edges (the KIH contacts). Graph theory is the robust mathematical framework built from this generic definition, and its applications emerge in diverse fields including operational research, genetics, linguistics, geography, sociology, architecture and many others (Wilson, 2010). In terms of applications to protein science, graph theory has been used in the form of Protein Structure Networks (Bhattacharyya *et al.*, 2016), for studying the rigidity of proteins (Sim *et al.*, 2015), probing the evolutionary constraints on amino-acid mutation (Parente *et al.*, 2015), comparing spatial arrangements of secondary structure elements (Grindley *et al.*, 1993), and representing pathways of protein–protein interactions (Huang *et al.*, 2014). Here, we apply tools from graph theory to address the problem of automatically classifying existing coiled-coil protein structures and partially enumerating the ‘dark matter’ of that protein structural space. We make particular use of the catalogue of graphs presented in the book ‘An Atlas of Graphs’ (Read and Wilson, 1998), and therefore refer to our system as the Atlas Classification. This is an updated catalogue of natural structures combined with an enumeration of some of the ‘dark matter’.

Since coiled coils are abundant, diverse, functionally important and amenable to protein design (Grigoryan *et al.*, 2009; Woolfson, 2005, 2017), they represent the ideal choice of protein substructure upon which to demonstrate the application of the Atlas Classification. To represent coiled coils as graphs, we have developed a Python-based implementation of the program SOCKET (Walshaw and Woolfson, 2001) for identifying KIH interactions, and therefore coiled coils, within protein structures. We call this interactive SOCKET, iSOCKET, due to the interactive computational tools it provides for analyzing and visualizing side-chain packing.

Combining the experimental coiled coils interpreted by iSOCKET and Atlas Classification yields the Atlas of Coiled Coils. This is an update of the PTCC, which contains coiled coils that were not present in the PDB when the PTCC was originally constructed. Moreover, the web-interface for the Atlas of Coiled Coils is interactive and allows the user to visually inspect the classification and, by adjusting geometric parameters, to probe the variation in helical packing across the PDB. We highlight the automatic identification and classification of a subset of previously unclassified coiled-coil structures, namely the α-helical barrels. Finally, our classification scheme shows regions of protein-structure space that are currently unoccupied, presenting a clear challenge to the next generation of protein-design studies.

## 2 Materials and methods

### 2.1 iSOCKET

At its core, iSOCKET follows a similar procedure for identifying knobs-into-holes (KIH) interactions to that described fully in the original SOCKET paper (Walshaw and Woolfson, 2001). Briefly, this proceeds as follows: Given a protein structure, the α helices are extracted and the centroid of the side-chain is stored for each residue. The helices are then considered in a pairwise manner. For each residue on the first helix, the four closest side-chain centres from the residues on the second helix are determined. If each of these four distances is less than a user-specified cutoff distance (the SOCKET cutoff, scut), then this is recorded as a KIH interaction with the residue on the first helix as the knob residue, and the four residues on the second helix forming the associated hole residues. The value of scut defines how tightly the knob must pack in the hole: reducing scut decreases or maintains the number of KIH interactions that are detected.

The parameter scut offers one method of filtering the KIH interactions that are detected. It is possible to filter further, for example, based on other geometric criteria or the amino acid composition of the packing and/or surrounding residues. The object-oriented nature of iSOCKET allows the user direct access to the KIH interactions and makes adding such criteria facile.

The core iSOCKET algorithm and associated convenience functions are available as the knobs_into_holes add-on module for ISAMBARD (Wood *et al.*, 2017), our recently-described open-source software package for the analysis and rational design of biomolecules (https://github.com/woolfson-group/isambard). iSOCKET builds on the AMPAL framework that ISAMBARD uses for representing biomolecules computationally, allowing seamless integration with its suite of analysis tools.

There are online tutorials are freely available as part of the web application source code (https://github.com/woolfson-group/isocket/wiki). These show the use of iSOCKET alongside ISAMBARD, introduces some of the convenience methods for probing individual KIH interactions in more detail, and demonstrate how to query the data used for the web application. The code for interpreting KIH interaction graphs in the context of the Atlas of Graphs is also represented in the tutorials.

A second add-on to ISAMBARD written for this study is the parmed_to_ampal module, which enables the parsing of mmCIF files into ISAMBARD, using the ParmEd library (https://github.com/ParmEd/ParmEd). This ensures that, unlike SOCKET, iSOCKET can be used to interpret the KIH packing within mmCIF files and therefore arbitrarily large structures.

### 2.2 Classification protocol

The initial set of PDB accession codes was taken from the latest update of CC+ (10 August 2016), filtered for canonical, non-redundant (70% redundancy cutoff) coiled coils containing at least 11 residues.

The expanded set was taken from the PDB on 23 November 2016. We filtered all the available structures to include all X-ray crystal structures with resolution ≤3 Å and used the option to omit large structures. The representative structures at 90% sequence identity were chosen, resulting in 35 476 accession codes.

For each code, the mmCIF file for the preferred biological unit [assigned using PISA (Krissinel and Henrick, 2007)] was downloaded from the PDBe and converted into an AMPAL object using tools in ISAMBARD. iSOCKET was used to find KIH interactions within protein structures, and to interpret this information in the form of a mathematical graph.

The Atlas Classification was implemented in Python, and made extensive use of the networkx module (Hagberg *et al.*, 2008). In particular, the graph_atlas_g method was used to generate the initial graph Atlas, and the is_isomorphic function to categorize graph pairs as being isomorphic.

## 3 Results

### 3.1 iSOCKET automatically identifies coiled coils

iSOCKET was conceived and written as an open-source Python-based application programming interface (API) for identifying and analyzing side-chain packing in protein structures. The main aims of iSOCKET were to allow non-expert users to analyze coiled-coil packing in an intuitive way, and to allow more-accomplished users direct access to the packing detail, and to perform geometric analyses on coiled-coil regions of interest in arbitrarily large protein structures.

As described in Section 2, the core algorithm for detecting knobs-into-holes (KIH) interactions is similar to that of the original SOCKET program (Walshaw and Woolfson, 2001). However, iSOCKET confers several advantages over the foregoing program. The original program required a user-defined distance cutoff for assigning KIH (default 7.0 Å), which were assigned in a binary fashion. The updated program collects all KIH at a deliberately large distance cut-off value (10 Å), and allows the user to select any threshold below this. This allows for a more-generous assignment of KIH interactions. Additionally, arbitrarily large structures can be analyzed, including complex coiled coils and larger structures containing multiple coiled coils, with both mmCIF and PDB files formats being handled. iSOCKET also enables analysis of the protein structural environment that surrounds each individual KIH interaction, thus making it a powerful tool for analyzing coiled-coil packing in detail. Within iSOCKET, convenience methods have been added for analyzing the packing geometry of individual KIH interactions in detail. Properties defined in detail elsewhere (Walshaw *et al.*, 2001; Walshaw and Woolfson, 2001, 2003), such as the knob-type, the depth of side-chain interdigitation, the core-packing angle, and the complementarity of the KIH interactions may all be calculated easily for both parallel and antiparallel coiled coils. Furthermore, the object-oriented basis of iSOCKET facilitates interpretation of KIH interaction networks, by representing these as a mathematical graph.

Importantly, and moving onto the main focus of this paper, representing coiled coils as graphs allowed them to be classified automatically following the Atlas Classification. To facilitate this, the α helices of the coiled coil form the nodes of the graph, and these are joined by edges that represent the KIH interactions. Since there may be many KIH interactions between a given pair of helices, there may be many edges joining two nodes on the graph. Each edge has a direction, starting from the helix that provides the knob residue and ending at that which provides the hole residues. Each node in the graph must have at least one edge associated with it, but the entire graph need not be connected. Indeed, where there are multiple separate coiled coils within the same protein structure these form the ‘connected components’ of the protein graph. The connected subgraphs are classified individually, since each represents exactly one coiled coil.

It is straightforward to represent the mathematical graph in the form of a simple diagram (Fig. 1D). Thus, via these KIH graphs, the visualization of coiled-coil interactions used in the PTCC, and beyond, can be automated.

### 3.2 The Atlas of Graphs is the basis of the automated classification scheme

**Fig. 2.**
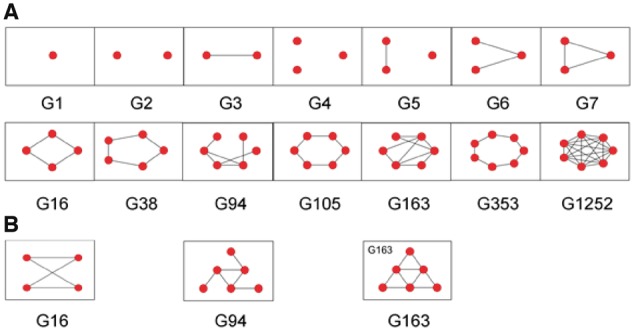
Small simple graphs from the Atlas of Graphs. (**A**) The first seven graphs (omitting the trivial graph G0) are shown in the top row, and a selection of later graphs are shown in the bottom row. These include the cyclic graphs G16, G38, G105 and G353. The graphs G2, G4 and G5 are disconnected. Each pair of nodes in G1252 is connected by an edge. (**B**) Isomorphs of G16, G94 and G163 are shown below their equivalents in (A) (Color version of this figure is available at *Bioinformatics* online.)

A simple graph contains no weighted or directed edges, and no edges that begin and end at the same node. The Atlas of Graphs (Read and Wilson, 1998) is an enumeration of all possible simple graphs with ≤ 7 nodes, which we refer to as small simple graphs. There are 1253 such graphs, which can be ordered by complexity and named accordingly (Read and Wilson, 1998). The trivial graph, containing no nodes and no edges, is named ‘G0’; ‘G1’ contains just one node; and the complete graph with 7 nodes and 21 edges (the maximum number possible) is named ‘G1252’ (Fig. 2A). It is theoretically possible to extend the Atlas to include larger simple graphs (Brinkmann *et al.*, 2013), although exhaustive enumeration rapidly becomes impractical as the number of nodes increases; for example, the number of distinct simple graphs with 17 nodes surpasses Avogadro’s number by a factor of more than 400. In our classification scheme, the coiled-coil graphs identified using iSOCKET are categorized according to their position in the Atlas of Graphs (Fig. 2A).

Graph theory not only provides enumeration, but also tools for comparison: two mathematically equivalent graphs are said to be isomorphic. The concept of the isomorphism underlines that it is the connectivity and not the spatial arrangement that defines the graph. In Figure 2B, the three graphs G16, G94 and G163 are represented; these are isomorphic to the graphs presented directly above them in Figure 2A. Any simple graph, with ≤ 7 nodes, is isomorphic to exactly one graph in the Atlas of Graphs and can be named accordingly. The difficulty of determining whether two graphs are isomorphic increases dramatically with the number of nodes and edges in the graphs. Indeed, the question of whether any arbitrarily chosen pair of graphs can be tested for isomorphism in polynomial time is an unsolved problem in computer science (Kobler *et al.*, 2012). For small simple graphs the problem is computationally facile.

**Fig. 3.**
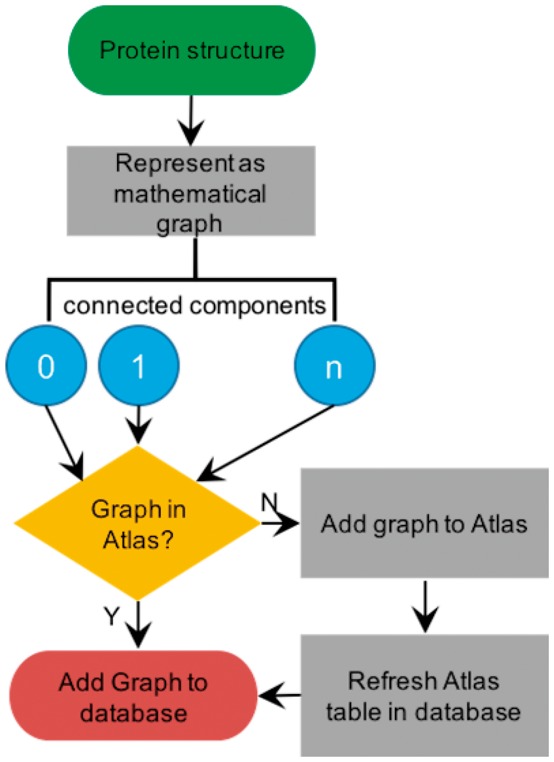
Procedure for classifying coiled coils. iSOCKET is used to identify graphs of KIH interactions within the protein structure and compare these, via isomorphism, to the graphs of the Atlas of Graphs. If no isomorph is found, the Atlas of Graphs is extended, and the new graph is added to the Atlas table in the database (Color version of this figure is available at *Bioinformatics* online.)

The procedure we used for classifying coiled coils is outlined diagrammatically in Figure 3, and is as follows: iSOCKET is used to find KIH interactions within a protein structure, which are then represented as a mathematical graph. This is converted to a simple graph and each of its connected components is classified separately (i.e. as individual coiled coils) *via* isomorphism to the Atlas of Graphs. With reference to Figure 2A, the graphs associated with a coiled-coil dimer, a hexameric barrel and the complex coiled coil in Figure 1 are ‘G3’, ‘G105’ and ‘G163’, respectively. A structure containing a separate dimer and trimer has two connected components: ‘G3’ and ‘G7’. Provided that the coiled coil contains ≤ 7 helices, its representative graph will be determined rapidly. For larger, complex coiled coils, we must confront the combinatorial explosion that prevents the Atlas of Graphs remaining exhaustive for larger graphs. Pragmatically, we introduce larger graphs as they are encountered: a graph that does not fit into the set of existing categories defines its own category and thus the Atlas of Graphs is expanded. Specifically, the graph corresponding to the first coiled coil to be encountered that contains > 7 helices is added and named as the previously unseen graph ‘U1’. The second such coiled coil is then either isomorphic to the first (and so belongs to the ‘U1’ category), or initiates another new category ‘U2’. All known coiled-coil structures can be categorized in this way.

### 3.3 The Atlas of Coiled Coils is an automated coiled-coil classification scheme

We formalized the notion that true coiled-coil packing is formed between pairs of helices that share multiple tightly packed KIH interactions, using a combination of two parameters (Fig. 1 and Section 2). First, the SOCKET cutoff (scut, Fig. 1E) defines the maximum distance between the centre of mass of a knob residue and those for each of the hole residues—this was set to 7.0 Å for the PTCC. Second, the knob cutoff (kcut, Fig. 1E) requires that there are more than kcut KIH interactions between each pair of helices defined to be associating in the coiled coil. For example, with scut = 7.0 Å and kcut = 2, each pair of interacting helices in the coiled coil must share 3 or more KIH interactions that pack more tightly than 7.0 Å.

To observe the effect of the values of these parameters on the coiled-coil classification, we classified each structure using values of scut between 7.0 and 9.0 Å at increments of 0.5 Å and of kcut between 0 and 3 at increments of 1. At each of the 20 combinations of these two parameter values, the iSOCKET graph representing the KIH packing was calculated, and the name of each of its constituent connected components determined. For a fixed cutoff pair, this yielded the coiled-coil composition of the structure.

Initially, we followed the above procedure for each structure in the CC+ database (Testa *et al.*, 2009), i.e. the set of structures that have already been identified by SOCKET as containing coiled coils. The resulting classification, the Atlas of Coiled Coils, serves as an automatically generated update to the PTCC (Moutevelis and Woolfson, 2009).

**Fig. 4.**
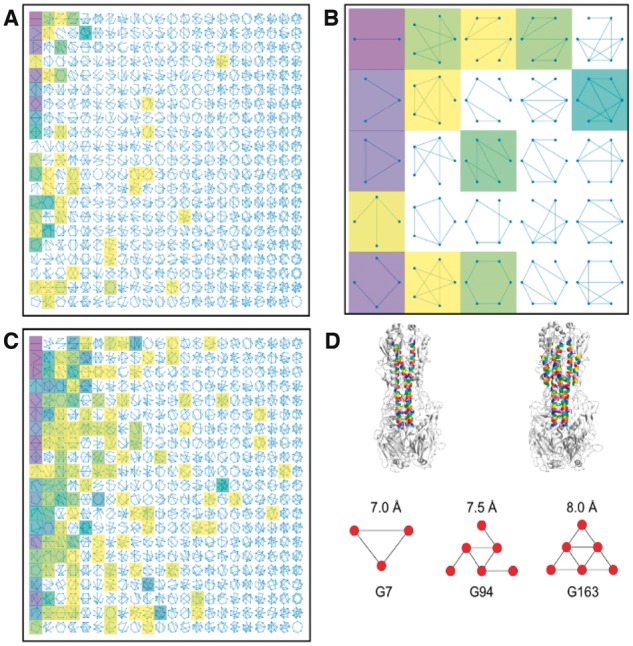
The Atlas of Coiled Coils. (**A**) Static image of the interactive visualization of the classification data. An array of cartoon representations of mathematical graphs is shown, each representing one category in the classification scheme. Categories that are populated at fixed values of scut (7.0 Å) and kcut (3) are highlighted with shaded boxes: darker shades correspond to larger numbers of extant structures. (**B**) Close-up of the 25 graphs in the top left corner of (A). (**C**) As in (A), but with scut = 9.0 and kcut = 0. (**D**) Top: The structure of hemagglutinin (4bsa) (Xiong *et al.*, 2013), with coiled-coil helices at 7.0 Å (left) and 8.0 Å (right) highlighted in colour. Images generated using PyMOL. Bottom: The associated coiled-coil graphs are shown at the indicated values of scut (kcut = 2 in each case) (Color version of this figure is available at *Bioinformatics* online.)

An interactive application that visualizes these classification data is freely available online (http://coiledcoils.chm.bris.ac.uk/atlas). Two static images of this are shown in Figure 4. The basis of the visualization is a grid showing cartoon representations of each of the 461 graphs from the Atlas of Graphs that is connected and satisfies the condition that all nodes have at most 4 incident edges. Each graph represents a category within the classification scheme. If a category is populated by a coiled coil its graph is highlighted with a shaded box, the colour of which relates to how densely the category is populated. For this, we used the viridis colour palette (https://matplotlib.org/examples/color/colormaps_reference.html), with darker colours representing more-densely populated categories. Unshaded graphs represent the unpopulated categories, i.e. the aforementioned structural ‘dark matter’ of coiled-coil-structure space.

In the online version, a mouse-over hover tool can be used to display the name of each graph, the number of corresponding coiled coils and the percentage of the total population that this represents. Sliders allow the user to filter the coiled-coil data dynamically and observe the resulting changes to the classification; i.e. how the number of observations within each category is affected. For example, Figure 4A shows the Atlas of Coiled Coils where scut = 7, kcut = 3. Here, 49 of the 461 categories shown are populated by at least one structure. However, many are populated by exactly one; there are just 14 distinct graphs for which there are more than 10 coiled-coil examples. Of these, only nine are present in the original version of PTCC (Moutevelis and Woolfson, 2009). The five ‘new’ forms include a natural extension of the first column of the PTCC to 5- and 6-helix ‘sheets’, as well as the graphs G94 and G163 (Fig. 4D), which are discussed below.

Other tools allow the user to zoom, resize and reset the image. Figure 4B shows a close-up of the visualization. The first column contains the four most-densely populated graphs; these are also heavily populated in the PTCC. The other highlighted graphs include G163 (second row, final column) and the cyclic graph for the hexameric barrel (bottom row, third column) for which there are currently 17 and 7 examples, respectively. There are no examples of hexameric barrels in the original PTCC: this highlights both the increase in structural data available, and the recent successes in designing α-helical barrel structures (Huang *et al.*, 2014; Thomson *et al.*, 2014; Zaccai *et al.*, 2011). Expanding on this, Figure 4A includes 4 examples of heptameric barrels, adding to the ‘slipped heptamer’ seven-helix coiled-coil in the PTCC (Liu *et al.*, 2006).

An arresting feature of the foregoing PTCC is that 74% of the structures are dimers. Furthermore, the five most common structural forms—dimer, trimer, tetramer, three- and four-helix sheets—represent 97% of coiled coils. The distribution in the new Atlas of Coiled Coils is similar with 62% dimers, and the five most densely populated categories (the first, second, third, fifth and seventh graphs in the first column) being the same as the PTCC and covering 84% of the coiled coils found.

Differences between the Atlas of Coiled Coils and the PTCC arise for two principal reasons: First, the number of coiled-coil structures available now is greater than when the PTCC was constructed (2905 versus 997), and so a larger number of sparsely populated categories is to be expected. Second, the Atlas classification was generated entirely automatically, and it is possible that the manual validation used to construct the PTCC would rule out some of the less densely populated categories as true coiled coils. Conversely, humans may be less adept at unambiguously identifying complex coiled coils, for example classifying only the central trimer over the surrounding entire assembly in the case of the six-helix bundles represented by G163.

Increasing scut or lowering kcut increased both the number and the variety of coiled coils detected (Fig. 4A and C). This was to be expected: as more KIH interactions were identified there was an increased likelihood of peripheral helices being included in more-complex coiled coils. Whilst the absolute number of dimers represented in Figure 4C (1831) was greater than in Figure 4A (1759), the proportion was reduced in the former to just 27%. At this highly permissive cutoff combination, loose packing between proximal helices is included and the resulting graphs may not represent tightly packed coiled coils. As the graphs get larger, the chances of two similar structures being placed into distinct categories increases, and so the specificity of the classification diminishes. As a counterpoint to this, there is greater sensitivity as many of the categories were more-densely populated. For example, there were 38 structures represented by G163. Manual inspection revealed these to be viral insertion proteins; this unsupervised classification scheme has grouped together structures of similar function, outside of its initial remit of coiled-coil classification. Strictly maintaining more-restrictive cutoffs would not group these structures in this way.

It is misleading to view a single combination of scut and kcut as being representative. To capture all the structures in the PTCC that correspond to small simple graphs for instance, scut must be varied between 7.0 and 7.5 Å, and kcut between 2 and 3. The graphical representation of an individual coiled coil may be sensitive to parameter values, as demonstrated in Figure 4D for a structure of hemagglutinin (PDB code: 4bsa) (Xiong *et al.*, 2013). Fixing kcut = 2 and setting scut = 7.0, 7.5 and 8.0 Å resulted in three different graphs. At low scut, only the central trimer was detected, but as it was increased, interactions were also found between the helices of the trimer and three outer helices. This pattern was common for the set of structures that were G163 for at least one of the 20 parameter combinations. The antiparallel six-helix bundle represented by G163 is a unifying feature of class 1 viral fusion proteins; these facilitate membrane fusion and therefore viral entry into host cells (Bosch *et al.*, 2003; Harrison, 2015; Kirchdoerfer *et al.*, 2016; Markosyan *et al.*, 2009). Central to this function is the large-scale conformational changes that switch the structure between an extended trimer and six-helix bundles. In this case, our structural classification scheme automatically grouped structures together that share a clear structure-function relationship.

The automated steps in the classification greatly reduced the challenge of expert manual validation: without it, classification would be impractical for the 2905 protein structures taken from CC+, and nearly impossible for the 35 476 nominal coiled-coil structures from the PDB.

### 3.4 iSOCKET and the Atlas identify and classify α-helical barrels

To demonstrate the utility of iSOCKET and the Atlas of Coiled Coils classification scheme, we searched for an emerging class of coiled coils of relevance to protein design, namely α-helical barrels (Thomson *et al.*, 2014; Woolfson *et al.*, 2012; Zaccai *et al.*, 2011). For this, we extended our classification scheme beyond the set of structures in CC+ to include a representative set of 35 476 structures from the PDB, selected as detailed in Section 2. These data can be selected to view via a drop-down menu in the interactive visualization.

**Fig. 5.**
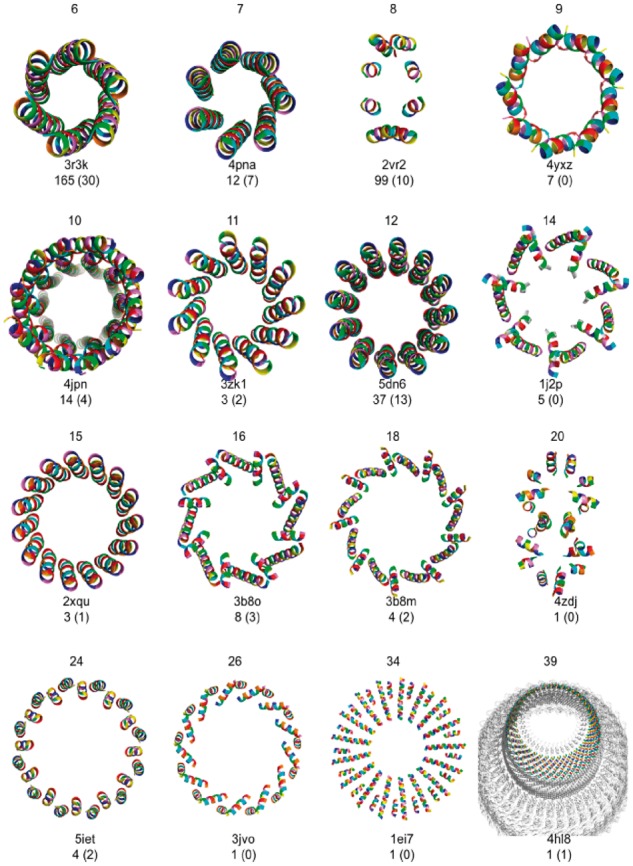
α-Helical barrels found by iSOCKET in CC+ and the PDB. Below each image, the PDB accession code of a representative structure is given along with the number of examples identified at any cutoff, and with scut ≤ 7.5 and kcut ≥ 2 in parentheses. Images generated using PyMOL (www.pymol.org) (Color version of this figure is available at *Bioinformatics* online.)

The larger α-helical barrels would have formed the top row of the PTCC, but the original SOCKET algorithm does not interpret coiled-coil-barrel assemblies that have more than 6 helices, instead detecting a series of dimers (e.g. 4pna). For iSOCKET, this limitation was corrected, and it interprets all barrels as cyclic graphs (Fig. 2A). All cycles from 3–7 are captured in the Atlas of Graphs and there are corresponding coiled-coil structures for each of these (Fig. 4A). Larger cycles are not part of the Atlas of Graphs and so barrels with more than 7 helices are not represented. However, our classification protocol (Fig. 3) updates the Atlas with larger graphs as they are encountered, and it is simple to extract the cyclic graphs from this set. The structures represented by these contain large helical barrels, Figure 5.

At looser cutoff values, 200 large barrels (≥7 helices) were detected, almost half of which (99) contained 8 helices. On closer inspection, many of these octamers including human dihydropyrimidinase (2vr2, depicted in Fig. 5) did not fit the intuitive notion of a barrel (i.e. cylindrical) shape, although the underlying graphs were cyclic. For the future, we aim to learn more about packing in α-helical barrels by investigating these examples in more detail. To date, the only oligomer states from 5 to 20 without example structures are 13, 17 and 19. These are the largest prime numbers in this range. This suggests that larger assemblies are unlikely to be formed other than as the composition of smaller repeating arrangements. The largest barrel, containing 39 helices, is in the 10 MDa vault ribonucleoprotein particle (4hl8) (Fig. 5).

## 4 Discussion

We have developed a Python-based API, iSOCKET, and used tools from graph theory to identify α-helical coiled coils automatically via their knobs-into-holes (KIH) interactions between partnering α helices (Crick, 1953; Walshaw and Woolfson, 2001, 2003), and to classify these into an Atlas of Coiled Coils.iSOCKET enables both expert and non-expert users to interrogate coiled-coil structures from assemblies down to atomistic level. The code is modular, extensible and open-source, and we encourage users to make their own modifications. We envision its adaptation for analyzing more-general knobs-into-holes interactions between different secondary structure elements (Fraga *et al.*, 2016).

Our classification provides an updated version of the Period Table of Coiled Coils (PTCC) (Moutevelis and Woolfson, 2009) and brings several advantages. Importantly, considering the exponential growth in protein structures deposited to the PDB, the classification is automatically generated and so is readily updateable. Visualization of the Atlas gives a simple overview of the classification (Fig. 4), depicting each category of coiled coil as a simple graph. Interactive tools allow straightforward adjustments of well-defined structural parameters used to identify the coiled coils. Relaxing these parameters identifies larger, more-complex structural forms: showing the continuum between tightly packed coiled coils and looser arrangements of helices. These changes may be relevant for protein structure, stability and function (Hulko *et al.*, 2006; Lupas and Bassler, 2017; Swain *et al.*, 2009). When classifying large, complex arrangements of helices, subtle differences between structures may result in them having different graphs and being categorized separately. By grouping large graphs that share properties such as having the same number of edges or containing a common subgraph, or using more than two structural parameters to filter further, useful automated meta classification layers could be implemented to tailor the classification.

The interactive tools allow structures to be dialled in or out of view based on the strength of coiled-coil interactions that make. Furthermore, extant coiled coils are shown in the context of all possible coiled-coil structures; i.e. alongside the ‘dark matter’ of coiled-coil space (Taylor *et al.*, 2009; Woolfson *et al.*, 2015). In this way, we see the small extent to which natural coiled coils, and the currently small number of designed structures, have sampled the available structural space. By contrast, in the PTCC dark-matter structures can only be inferred by their absence. Other than for simple cyclically symmetric structures (in effect, the top row of the PTCC) this is difficult using the PTCC, and entirely impractical to do systematically. Focusing on the expanded set of these cyclic graphs for example yields the first formal classification of α-helical barrels (Fig. 5), further demonstrating the utility of the automated scheme. This Atlas of α-helical barrels presents a clear set of targets for protein designers (Huang *et al.*, 2014; Thomson *et al.*, 2014; Woolfson *et al.*, 2015; Zaccai *et al.*, 2011). This is not restricted to soluble proteins: one of the octameric regions identified is from Wza (2j58) (Dong *et al.*, 2006), that we recently used to guide the design of a membrane-spanning α-helical peptide barrel (Mahendran *et al.*, 2017). Classifying membrane proteins using our scheme yields an Atlas of transmembane helix packing (Niitsu *et al.*, 2017). These are challenging but potentially useful targets for design in bionanotechnology and synthetic biology (Joh *et al.*, 2014).

Another possible use of the Atlas of Coiled Coils is that by highlighting the unoccupied parts of coiled-coil-structure space, it provides clear targets and directions for these to be explored either through bioinformatics studies of sequence and structural databases, or via rational *de novo* design. Both of these will be challenging because, by definition, there are no examples to seed searches or to provide design principles, such as sequence-to-structure relationships needed to guide rational design (Woolfson, 2005, 2017). However, exploring this so-called ‘dark matter’ of coiled-coil space should be aided by recent developments in modelling and, specifically, in parametric protein design (Grigoryan and Degrado, 2011; Huang *et al.*, 2014; Parmeggiani *et al.*, 2015; Thomson *et al.*, 2014; Wood *et al.*, 2014, 2017). In this way, it is now possible to build models and optimize sequences for new coiled-coil structures. The ‘dark-matter’ graphs from the Atlas that are themselves composed of graphs for which there are existing structures, and that do not have any nodes with more than three incident edges, represent the best starting candidates (Boyken *et al.*, 2016). The explicit ‘dark matter’ in the Atlas of Coiled Coils highlights the scale of the challenge faced by protein designers, but also, we hope, provides some inspiration.

Finally, it is important to note that once a structure has been interpreted as a graph, the classification protocol that follows is entirely generic; i.e. it is independent of the of the secondary structure type(s) that the structure comprises (Fig. 3). Indeed, the nodes need not be secondary structure elements at all: they could be domains, or chains within a larger protein complex. To classify another protein architecture (or indeed, anything else) it is only this first step—explicitly defining the nodes and edges and therefore the conversion into graphs—that needs to be re-implemented. Since the Atlas Classification scheme is not exhaustive for graphs with >7 nodes, it is most useful where this limit is not frequently exceeded. It could be applied, for example, to the categorization of β-strands interacting via hydrogen bonds or protein–protein interactions identified by mutually buried surface areas.
